# The Approach to Sample Acquisition and Its Impact on the Derived Human Fecal Microbiome and VOC Metabolome

**DOI:** 10.1371/journal.pone.0081163

**Published:** 2013-11-18

**Authors:** Robin D. Couch, Karl Navarro, Masoumeh Sikaroodi, Pat Gillevet, Christopher B. Forsyth, Ece Mutlu, Phillip A. Engen, Ali Keshavarzian

**Affiliations:** 1 Department of Chemistry and Biochemistry, George Mason University, Manassas, Virginia, United States of America; 2 Department of Environmental Science and Policy, George Mason University, Manassas, Virginia, United States of America; 3 The Microbiome Analysis Center, George Mason University, Manassas, Virginia, United States of America; 4 Division of Digestive Diseases and Nutrition, Department of Medicine, Rush University Medical Center, Chicago, Illinois, United States of America; 5 Department of Biochemistry, Rush University Medical Center, Chicago, Illinois, United States of America; 6 Department of Pharmacology, Rush University Medical Center, Chicago, Illinois, United States of America; 7 Department of Molecular Biophysics and Physiology, Rush University Medical Center, Chicago, Illinois, United States of America; 8 Division of Pharmacology, Utrecht Institute for Pharmaceutical Sciences, Faculty of Science, Utrecht University, Utrecht, The Netherlands; Howard University, United States of America

## Abstract

Recent studies have illustrated the importance of the microbiota in maintaining a healthy state, as well as promoting disease states. The intestinal microbiota exerts its effects primarily through its metabolites, and metabolomics investigations have begun to evaluate the diagnostic and health implications of volatile organic compounds (VOCs) isolated from human feces, enabled by specialized sampling methods such as headspace solid-phase microextraction (hSPME). The approach to stool sample collection is an important consideration that could potentially introduce bias and affect the outcome of a fecal metagenomic and metabolomic investigation. To address this concern, a comparison of endoscopically collected (*in vivo*) and home collected (*ex vivo*) fecal samples was performed, revealing slight variability in the derived microbiomes. In contrast, the VOC metabolomes differ widely between the home collected and endoscopy collected samples. Additionally, as the VOC extraction profile is hyperbolic, with short extraction durations more vulnerable to variation than extractions continued to equilibrium, a second goal of our investigation was to ascertain if hSPME-based fecal metabolomics studies might be biased by the extraction duration employed. As anticipated, prolonged extraction (18 hours) results in the identification of considerably more metabolites than short (20 minute) extractions. A comparison of the metabolomes reveals several analytes deemed unique to a cohort with the 20 minute extraction, but found common to both cohorts when the VOC extraction was performed for 18 hours. Moreover, numerous analytes perceived to have significant fold change with a 20 minute extraction were found insignificant in fold change with the prolonged extraction, underscoring the potential for bias associated with a 20 minute hSPME.

## Introduction

Recent animal and human studies have highlighted the importance of the microbiota in maintaining a healthy state as well as promoting disease states, including not only gastrointestinal diseases but also chronic systemic metabolic and inflammatory diseases [1]. The role of the intestinal microbiota in regulating metabolism as well as intestinal and systemic immunity is now well established [2,3]. The intestinal microbiota exerts its profound physiological and pathological effects primarily through its metabolites, and not surprisingly metabolomics investigations have begun to evaluate the diagnostic and health implications of volatile organic compounds (VOCs) isolated from human feces [4–8]. 

Related by their volatility at ambient temperatures, the VOCs comprise a large and structurally diverse family of carbon-based molecules, of both natural and man-made origin. Specialized sampling methods, such as headspace solid-phase microextraction (hSPME), have greatly enabled the isolation of VOCs from a wide array of biological samples [9–12], including feces [4–8,13]. hSPME typically involves the partitioning of the VOCs from the headspace above a sample into a polymeric sorbent adhered to a fused silica rod (fiber), subsequent desorption of the VOCs into the heated inlet of a gas chromatograph, separation of the VOC mixture by gas-liquid partition chromatography, and detection by mass spectrometry. Spectral comparison to a reference database enables VOC identification.

The approach to stool sample collection is an important consideration that could potentially introduce bias and profoundly affect the outcome of a fecal metagenomic and metabolomic investigation. *In vivo* sample collection is generally desirable, as the resulting microbiome/metabolome is then a reflection of the native biological context, without potential *ex vivo* effects. However, to date, human fecal VOC investigations have typically examined samples collected after passage, allowing the stool to become exposed to the ambient environment. Since the descending and sigmoid colon are predominantly anaerobic, with the resident bacteria primarily comprised of obligate anaerobes [14,15], there is the potential that *ex vivo* bacterial metabolism, occurring between the time of passage and the freezing of the stool, may significantly alter the composition of the fecal VOC metabolome. While home stool collection is undoubtedly easier, far more feasible, and more economical to perform, it remained unclear if significant differences in fecal composition would appear if the samples were alternatively collected directly from the sigmoid lumen and immediately snap frozen to avoid any *ex vivo* effects. This information is critical for interpretation of stool metabolomics results, particularly before stool VOCs can be reliably used as diagnostic and risk stratification tools and/or exploiting stool VOC data to better understand the role of intestinal microbiota in the pathogenesis of gastrointestinal and systemic diseases. 

Potential sampling bias might also be introduced by the approach to the hSPME technique. Fiber choice and extraction duration are two prime considerations when performing a hSPME analysis of feces. While numerous fiber types are commercially available, the polarity of the targeted analytes generally dictates fiber selection [12]. As the fecal VOC metabolome is chemically diverse, a rational combination of several different sorbent chemistries is essential for a global metabolomic analysis of all the indigenous analytes [8]. Regardless of the sorbent type employed, the fecal VOC extraction profile is hyperbolic, with short duration extractions (e.g. 20 minutes) more susceptible to variable analyte titers as a consequence of subtle deviations in extraction duration [8]. While several fecal VOC investigations have utilized short hSPME durations [4–7], a quantitative hSPME analysis is ideally performed when the analyte distribution is in equilibrium between the sample and the fiber coating, during the plateau of a hyperbolic extraction profile (i.e. 16-18 hrs for fecal VOC extraction) [8,16]. It remains unclear if metabolome differences observed using short extraction durations simply reflect sampling bias due to the innate variability of the hSPME technique more so than variability in metabolite abundance among the samples. It is noteworthy however, that for some metabolites, titers plateau then subsequently wane with prolonged extraction duration [8], a phenomenon attributed to higher affinity compounds displacing those with lower affinity for the fiber, thereby lowering the titer of the latter [17].

We describe here a comparative microbiome and VOC metabolome analysis of fecal samples collected directly from the sigmoid lumen (via un-prep sigmoidoscopy) then frozen right away with those collected after passage (at home) and then frozen after a period of time. Additionally, we compare the derived VOC metabolome obtained using 20 min and 18 hr hSPME durations.

## Materials and Methods

### Fecal samples

This investigation was approved by the Institutional Review Boards at George Mason University and Rush University Medical Center and conducted after an informed, written research consent was signed by all study participants. Fecal samples were collected from 17 healthy subjects (a total of two samples were obtained from each subject, each sample collected on separate visits to the hospital, as detailed below). Table 1 depicts the demographic characteristics of the study subjects. Each subject completed a detailed health questionnaire that showed that none had any chronic GI or systemic disease, none had any GI symptoms, none were taking any regular medication except for blood pressure and cholesterol, and none used supplements including probiotics or prebiotics. No subject took antibiotics, for at least three months, and none were excessive drinkers of alcohol (less than 2 drinks per sitting per day for women and less than 4 drinks for men). The study participants were instructed not to change their usual dietary consumption and, as verified by dietary questionnaire, all participants had no change in their typical diet or health status between the two stool collections. Each subject had a stool collection on two occasions: once during sigmoidoscopy, Visit 1, and another time at home, within 24 hrs prior to Visit 2. The interval between the study subject’s two stool collections was never more than 7 days (Table 1). 

**Table 1:** Characteristics of the study participants.

**Healthy Controls N=17**	**Male**	**Females**
**Gender**	9	8
**Race**	5 Whites; 3 Blacks; 1 Asian	2 Whites; 5 Blacks; 1 Hispanic
**Age Range**	20-60	29-63
**Age Mean**	39.4	39.5
**BMI Range**	19.60-35.40	26.60-45.40
**BMI Mean**	26.24	35.71
**Currently Smoking During Time of Study (1-2 packs per day)**	2 out of 9	4 out of 8
**Days Between Collection Range**	1-7	5-7
**Days Between Collection Mean**	4.9	6.8

For endoscopy stool collection, Visit 1, each study subject underwent a limited un-sedated sigmoidoscopy after an informed, written consent. There was no colon preparation prior to sigmoidoscopy. The stool in the lumen of distal sigmoid was grabbed by a Roth Net (Ref 00711052; US Endoscopy, Mentor, OH) and removed with the sigmoidoscope. From the sigmoidoscope, the stool was then placed in a cryovial and placed in liquid nitrogen in the endoscopy room. Upon removal from the liquid nitrogen, the cryovial was immediately stored in a -80 °C freezer until analysis. At the completion of the study subject’s sigmoidoscopy, each subject was given a home stool collection kit to be returned to the hospital at Visit 2. 

For home stool collection, study subjects were provided with the supplies and instructions that informed them on how to put their stool into a BD Gaspak EZ Anaerobe Gas Generating Pouch System with Indicator (Ref 260683; Becton, Dickinson and Company, Sparks, MD) in order to minimize the exposure of stool to high oxygen ambient atmosphere. Subjects were asked to have a bowel movement, within 24 hours of Visit 2, to keep the sealed anaerobic stool bag in a cold environment, and to bring the anaerobic stool bag to the hospital. The stool was then immediately stored in a -80 °C freezer. The interval between passage of stool and storage at -80 °C was within 12 to 24 hours.

### Microbiome analysis

We interrogated the microbial taxa associated with the gut fecal microbiome using multi-tag pyrosequencing (MTPS). This technique allows the rapid sequencing of multiple samples at one time, yielding thousands of sequence reads per sample [18]. Specifically, we have generated a set of 96 emulsion PCR fusion primers that contain the 454 emulsion PCR linkers on the 27F primer (5’-AGAGTTTGATCCTGGCTCAG-3′) and 355R′ (5′-GCTGCCTCCCGTAGGAGT-3′) and different eight-base “barcode” between the A adapter and the 27F primer. Thus each fecal sample was amplified with unique barcoded forward 16S rRNA primers, and then up to 96 samples were pooled and subjected to emulsion PCR and pyrosequenced using a GS-Junior pyrosequencer (Roche). Data from each pooled sample were “deconvoluted” by sorting the sequences into bins based on the barcodes using custom PERL scripts. Reads were filtered based on quality scores (>30 quality units assigned by the 454) and length (>180 bp). Thus we were able to normalize each sample by the total number of reads from each barcode. We have noted that ligating tagged primers to PCR amplicons distorts the abundances of the communities, and thus it is critical to incorporate the tags during the original amplification step [18]. We identified the taxa present in each sample using the Bayesian analysis tool in Version 10 of the Ribosomal Database Project (RDP10). The abundances of the bacterial identifications were then normalized using a custom PERL script, and taxa present at >1% of the community were tabulated. We chose this cutoff because 2,000 reads per sample will only reliably identify community components that are >1% in abundance, while acknowledging that those less than 1% may indeed have significant biological and clinical relevance. A Pearson (n) principal component analysis was performed using the taxa abundance table and the statistical package XLSTAT 2012.6.02. Pearson’s correlation coefficients were calculated using Microsoft Excel. Unsupervised hierarchical clustering and heatmap generation was performed using the statistical package R (http://www.r-project.org/) using the Ward method and Pearson correlation for the distance measure.

### hSPME procedure

Aliquots (0.2 g) of each frozen fecal sample were dispensed into 4 mL WISP style screw thread amber glass vials, sealed with Black Top Hat PTFE/Silicone caps (J.G. Finneran, Vineland, NJ), and stored at -80 °C until analyzed. The following three SPME fibers (Supelco, Bellefonte, PA) were used in our investigation: 75 μm carboxen-polydimethylsiloxane (CAR-PDMS), 85 μm polyacrylate (PA), and 50/30 μm divinylbenzene (DVB)-CAR-PDMS. All fibers were preconditioned before use, as per the manufacturer’s instructions. All analyses were performed in duplicate. Sample vials were heated to 60 °C for 30 minutes then the hSPME fiber was positioned into the headspace above the feces and the fiber exposed to the volatiles for 20 minutes or 18 hours. The sample vial temperature was held at 60 °C for the duration of the exposure. The fiber assembly was then placed into the GC inlet for thermal desorption of the analytes.

### GC-MS Instrument

Samples were analyzed using an Agilent 7890A GC equipped with a DB5-MS capillary column (Agilent, Palo Alta, CA; 30 m length, 0.25 mm ID, and 0.25 μm film thickness), a 0.75 mm ID SPME injection port liner, and a 5975 inert XL mass selective detector (MSD) with triple axis detector. The GC injection port was operated in splitless mode at select inlet temperatures, dependent upon the SPME fiber used (300 °C, CAR-PDMS; 280 °C PA; 270 °C DVB-CAR-PDMS). Helium carrier gas was set to a flow rate of 1.17 mL/min. The GC oven was held at an initial temperature of 35 °C for 1 min, ramped at 3 °C/min to 80 °C, then 10 °C/min to 120 °C, and finally 40 °C/min to 260 °C, where the temperature was held for 1.5 min. The total run time for the analysis was 25.0 min. The MSD was scanned from 30 to 550 amu at a rate of 2.81 scans/sec.

### Data processing and analysis

VOCs were identified in the GC-MS chromatograms using the National Institute of Standards and Technology (NIST, Washington, DC) Automated Mass Spectral Deconvolution and Identification System (AMDIS, ver. 2.69) software and mass spectral library (NIST08). Only compounds with 85% or greater probability of match to a molecule in the NIST08 library were considered. Each AMDIS outfile, containing a list of identified metabolites and their corresponding peak height values, was filtered by custom Perl scripts designed to remove background analytes (e.g. siloxanes) and eliminate metabolite redundancies (retaining the replicate with the highest peak value). Duplicate sample data sets were combined by merging their AMDIS outfiles and averaging the corresponding peak height values. A comprehensive, three-fiber metabolite dataset was prepared for each sample by pooling the metabolites obtained using the CAR-PDMS, PA, and DVB-CAR-PDMS fibers and summing the corresponding peak height values (a peak height of zero was imputed for missing metabolites). A Perl script was then used to assemble a complete metabolite matrix containing all of the endoscopy and passaged home collected samples and their accompanying metabolites. Metabolites present in ≤20% of the samples were treated as one-offs and were removed. The metabolite matrix was arranged into two cohorts (an endoscopy cohort and a passaged home collected cohort) and outlier peak height values were identified in each cohort using a plot of (mean-median)/median for each analyte and a cutoff value ≥1.5. Outliers were replaced with the median value for that metabolite within the cohort. Metabolite peak height values were then standardized across the two cohorts by conversion to Z-scores (peak height-mean/standard deviation). A Pearson (n) principal component analysis was then performed using the standardized metabolite matrix and the statistical package XLSTAT 2012.6.02. Pearson’s correlation coefficients were calculated using Microsoft Excel. Unsupervised hierarchical clustering and heatmap generation was accomplished using the R statistical package and the Ward method and Pearson correlation for the distance measure. Fold change analysis was performed using MetaboAnalyst2.0 [19]. Custom Perl scripts were used to combine and compare the cohort metabolites to identify common and unique metabolites and to group the metabolites and their relative abundance into defined chemical classes. Bar graphs were prepared using GraphPad Prism ver. 4.0. 

## Results and Discussion

To ascertain if the fecal composition is affected by the approach to sample acquisition, we obtained 34 stool samples from 17 healthy volunteers (two samples from each volunteer were collected, one *in vivo* via endoscopy and another *ex vivo* by home collection after passage) and the samples were compared in terms of their microbiome and VOC metabolome.

**Figure 1:**
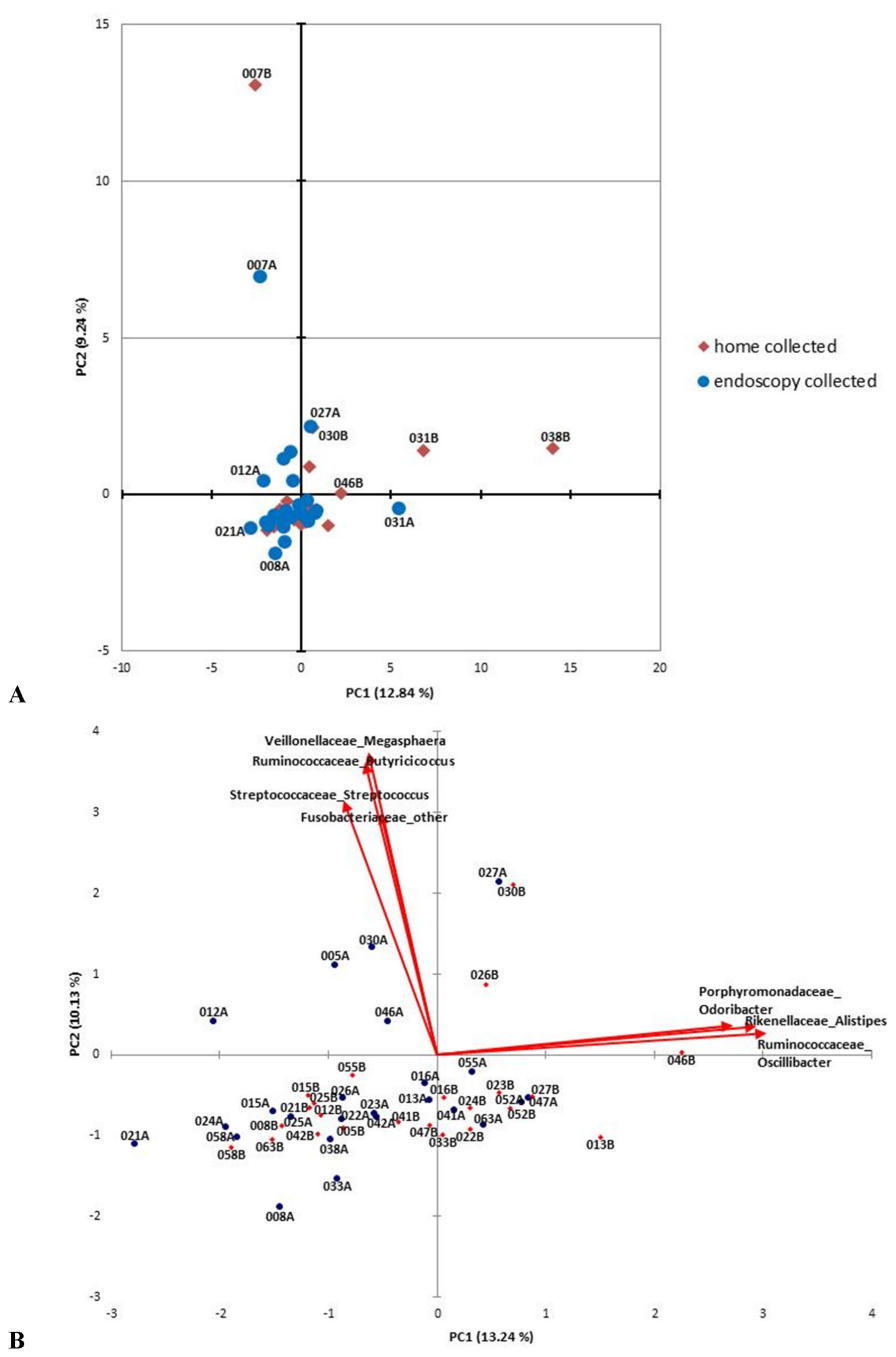
Principle component analysis of the microbiomes identified in the home passage and endoscopy collected human fecal samples. A) PCA plot derived from the identified taxa and their abundance. The first (PC1) and second (PC2) principle components are shown, which represent the two largest contributions to variation among the samples. B) Biplot containing a magnified view of the clustered region seen in A). The taxa that impart the greatest contribution towards the two principle components are indicated, with vectors indicating the magnitude and direction of the factor loadings. While the two cohorts generally appear clustered in A), a pairwise comparison of matched home passage and endoscopy collected samples in B) reveals some variation in the derived microbiomes. Home collected samples (red diamonds) have a name designation containing the letter B. Endoscopy collected samples (blue circles) have a name designation containing the letter A. Matched samples have the same number designation. See text for further discussion.

Using multi-tag pyrosequencing, the microbiome composition of the fecal samples was determined and the identified taxa were compared among the samples. A total of 50 different taxa (22 families, 50 genera) were identified in the feces. Figure 1 shows the distribution of the samples along the first two axes of a principal component analysis (PCA). As seen in Figure 1A, among the two cohorts as a whole, the majority of the samples tightly cluster on the PCA plot, indicative of relatively small variance in the overall microbiome composition (in contrast to the metabolome, in which the two cohorts are well segregated, as discussed below). A pairwise comparison of matched home passage and endoscopy collected samples reveals that individual pairs demonstrate some degree of variance in their microbiome composition (Figure 1B), with *Ruminococcaceae Oscillibacter*, *Rikenellaceae*, and *Porphyromonadaceae* having the largest contribution to the first principle component and *Veillonellaceae*, *Ruminococcaceae Butyricicoccus, Streptococcaceae Streptococcus*, and *Fusobacteriaceae* contributing the most variance to the second principle component, although with no apparent bias towards either fecal isolation technique. Pearson’s correlation coefficients (r), calculated by comparison of the microbiome content in the matched fecal samples, illustrate that while none of the paired samples are identical, 39% of the matched sample pairs demonstrate a very strong correlation (r>0.9), 42% demonstrate a strong correlation (0.7<r<0.9), and 19% display weak to moderate correlation among their microbiomes (0.2<r<0.7) (Figure S1). Thus, the majority (81%) of the home collected and corresponding endoscopy collected samples have strong to very strong correlations in their microbiome content (0.7<r<0.99). Additionally, unsupervised hierarchical clustering analysis does not significantly differentiate the microbiomes derived from the home collected and endoscopy collected fecal samples (Figure S2). Collectively, considering the degree of similarity observed in the derived microbiome content among the home and endoscopy collected samples, the significant effort and expense associated with the endoscopic collection of stool may not be justified for a metagenomics investigation of human feces.

To determine if the VOC metabolome is affected by the fecal collection technique, VOCs from the endoscopy and passage collected samples were extracted by hSPME then identified by GC-MS. To ensure greater metabolome coverage while still accommodating reasonable throughput, 3 different hSPME fiber types were chosen for this analysis (CAR-PDMS, PA, and DVB-CAR-PDMS). Whenever possible (sample abundance permitting), the extractions were performed in duplicate (using different aliquots) and the replicates combined by averaging chromatographic peak height values. As the fecal VOC extraction profile is hyperbolic, we elected to perform both 20 minute and 18 hour extractions of each sample (using different aliquots), to permit a comparison of the results. Hence, a total of 408 chromatograms were acquired from the 34 participant fecal samples. 

Utilizing a 20 minute hSPME, a combined total of 1371 different VOCs were identified in the endoscopy collected cohort. Similarly, when extracted for an identical duration, the home passage collected cohort contains 1404 total analytes, a difference of only 33 analytes relative to the endoscopy group. As anticipated, given the hyperbolic nature of the fecal VOC extraction profile [8], an 18 hour extraction isolates considerably more VOCs; 2097 total metabolites are associated with the endoscopy cohort and 2190 are found in the home passaged group (a difference between the cohorts of only 93 metabolites). Overall, regardless of the extraction duration used, it is apparent that both approaches to fecal collection yield a similar number of total VOCs.

**Figure 2:**
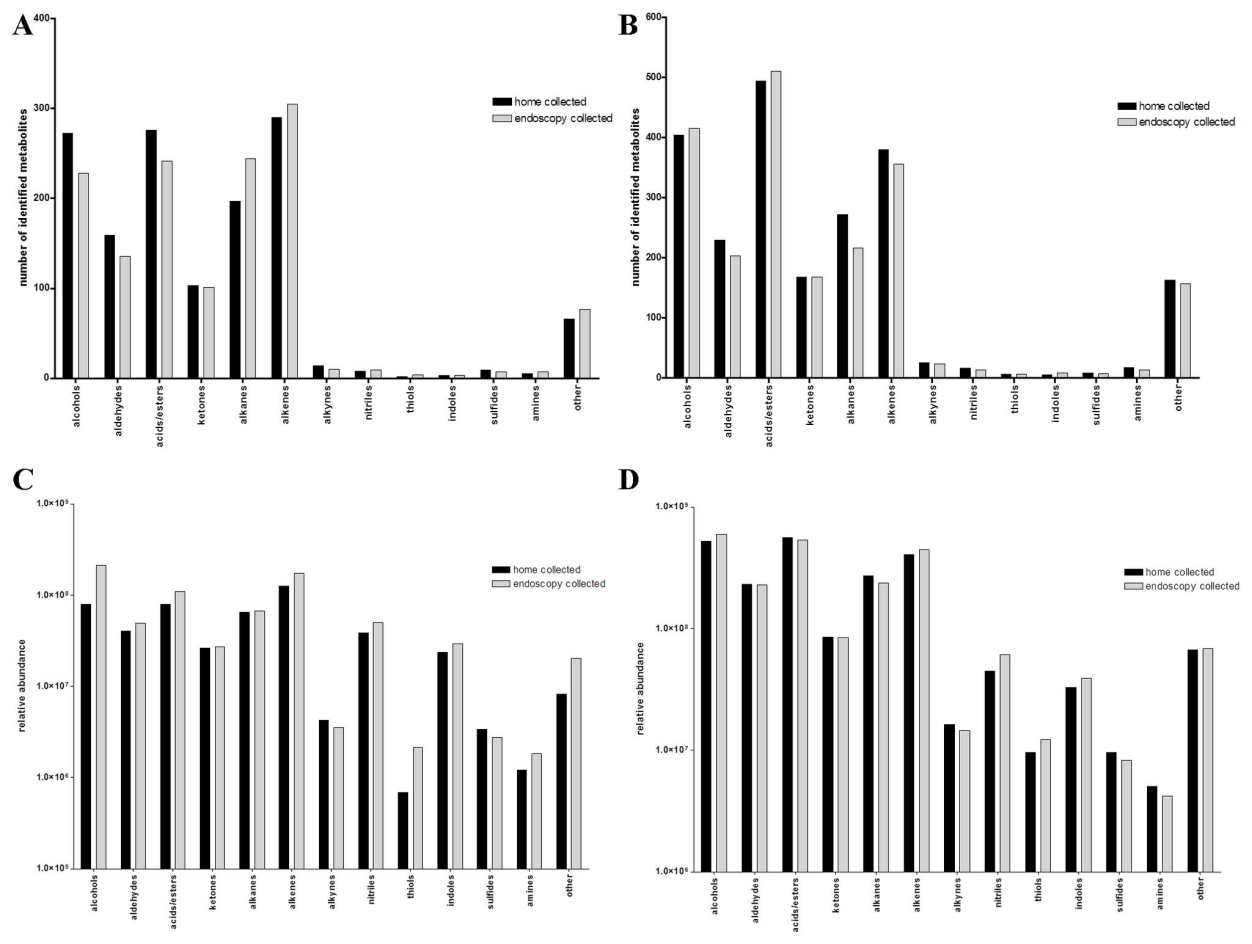
Cohort metabolite composition and abundance. The pooled analytes present in each cohort were distributed among the indicated chemical classes and then tallied. A and B) The graphs indicate the total number of analytes in each class for the 20 minute hSPME (A) or the 18 hour hSPME (B) metabolomes. The sum of the standard deviation across the chemical classes is 134 for the 20 minute data and 108 for the 18 hour data, emphasizing the greater similarity between the cohorts in the latter. C and D) The relative abundance (peak height) of the analytes present in each cohort were distributed among the indicated chemical classes and then summed. The graphs indicate the relative abundance of each class for the 20 minute hSPME (C) or the 18 hour hSPME (D) metabolomes. Although the cohort similarity is apparent regardless of extraction duration, the sum of the coefficient of variation across the chemical classes totals 3.5 for the 20 minute data and 1.1 for the 18 hour data, highlighting the greater similarity between the cohorts in the latter.

Figure 2 compares the composition of the cohorts in terms of the number of identified analytes and the relative abundance in each of the indicated chemical classes. With the 20 minute extraction duration, the overall chemical distribution appears quite similar among the two collection techniques (Figure 2A), with a slight bias towards oxidized metabolites in the home passaged cohort (alcohols, aldehydes, acids/esters) and reduced metabolites in the endoscopy cohort (alkanes, alkenes). With an 18 hour hSPME, the similarities among the cohorts are even more pronounced (Figure 2B), implicating the variability associated with short hSPME durations [5] as a primary contributor to the variability observed between the 20 minute metabolomes (Figure 2A). A similar trend is also observed when comparing the relative analyte abundance (Figures 2C and 2D); specifically, greater variability associated with the 20 minute hSPME, yet a very similar distribution of metabolite abundance among the chemical classes, whether the feces is isolated *in vivo* by endoscopy or *ex vivo* using a home passage collection technique. 

Although the overall chemical milieu appears similar between the two cohorts (based on the comparisons above), noteworthy differences arise when comparing the specific composition of analytes within each of the chemical classes. Figure S3A presents the similarities and differences within each of the chemical classes, comparing the 20 minute metabolomes. While a significant number of metabolites are common to both of the cohorts, in most of the chemical classes a substantial number are uniquely associated with either the endoscopy or home collected samples. Equivalent results are obtained when comparing the 18 hr metabolomes (data not shown). While these metabolomic differences between cohorts could be a reflection of the fecal collection technique, the ‘cohort-unique’ metabolites are more likely attributed to variations in dietary intake, as nearly all of these unique analytes appear in only a small proportion (20% or fewer) of the stool samples analyzed (Figure S4). When these low frequency metabolites are omitted, the metabolome composition among the cohorts appears nearly identical (Figure S3B and S3C and Table 2). One notable exception, 1,3-bis(1,1-dimethylethyl)-benzene, is exclusively linked to the endoscopy collected fecal samples and occurs at a high frequency among those samples, in both the 20 minute and 18 hour metabolomes (Table 2). A radiolysis product of polypropylene, 1,3-bis(1,1-dimethylethyl)-benzene is generated during gamma irradiation sterilization of microcentrifuge tubes [20], the same type of tubes we used for storing the endoscopy collected samples (the home passage collected samples were placed in different types of tubes). Thus, 1,3-bis(1,1-dimethylethyl)-benzene is most likely an exogenous analyte derived directly from the plasticware. Methyl- and propylparaben are two additional analytes also found uniquely associated with the endoscopy collected samples (Table 2). Present as preservatives in lubricating jelly, it is probable that these two exogenous compounds were introduced when lubricant became unintentionally incorporated into a subset (44%) of the fecal samples, during the endoscopic collection of stool (the parabens were also detected in 15% of the endoscopic samples with a 20 minute hSPME). 

**Table 2:** Unique metabolites associated with the home passage collected and endoscopy collected fecal samples.

**20 Minute Extraction**			**18 Hour Extraction**		
**Metabolites Identified as Unique to the Home Passage Collected Cohort**		**Frequency (%)**	**Metabolites Identified as Unique to the Home Passage Collected Cohort**		**Frequency (%)**
acids/esters	Butanoic acid, 2-methylpropyl ester*	31	acids/esters	1,2-Benzenedicarboxylic acid, diphenyl ester	31
	Benzenepropanoic acid, methyl ester*	25	alkenes	Benzene, 1-ethynyl-4-methyl-	25
alkenes	Benzene, 1-ethynyl-4-methyl	31	aldehydes	2-Nonenal	31
	1,1’-biphenyl, 4-methyl	46	other	Benzofuran, 2,3-dihydro-	25
other	undecanoic γ-lactone*	25		Acetylpyrazine	31
**Metabolites Identified as Unique to the Endoscopy Collected Cohort**		**Frequency (%)**	**Metabolites Identified as Unique to the Endoscopy Collected Cohort**		**Frequency (%)**
acids/esters	Acetic acid, 4-methylphenyl ester*	25	acids/esters	Benzoic acid, 3-hydroxy-, methyl ester	25
ketones	Cyclohexanone, 5-methyl-2-(1-methylethyl)-*	25	alkenes	Benzene, 1,3-bis(1,1-dimethylethyl)-	100
alkanes	Bicyclo[2.2.1]heptane, 2-chloro-2,3,3-trimethyl-*	31	other	Methylparaben	44
alkenes	Benzene, 1,3-bis(1,1-dimethylethyl)-	92		Propylparaben	25
				Quinazoline, 4-methyl	25

Several analytes (~30) deemed unique in the 20 minute metabolomes were found to be common to both cohorts after 18 hour extraction, and were thus excluded from the list (presumably a consequence of incomplete extraction with the shorter extraction duration). The inverse scenario was not found to occur (99% of the total metabolites compared among the 18 hour metabolomes appear common to both cohorts (Figure S3C), only half of which were detected with a 20 minute extraction duration). Metabolites highlighted with an asterisk are also uniquely associated with the same cohort after an 18 hour extraction, but appeared in less than 20% of the total number of samples in the 18 hour data set, so are not listed in the 18 hour column. Competitive dissociation by higher affinity analytes may account for the absence of these metabolites with prolonged extraction [17]. The frequency is the percentage of cohort members containing this metabolite.

Given their relatively low frequency of appearance, and considering their known affiliations with food, the unique cohort associations of 1-ethynyl-4-methylbenzene (a contaminant of cow’s milk [21]), as well as 5-methyl-2-(1-methylethyl)-cyclohexanone and undecanoic γ-lactone (two well-known phytochemicals with diverse taxonomic distribution [22–29]), may simply reflect dietary variations among the study participants, more so than alterations in the metabolome due to fecal collection technique. This may also be true for 4-methyl-quinazoline, uniquely detected in 25% of the 18 hr endoscopy collected samples (Table 2). While it is a known bacterial metabolite [30–32] derived from the shikimate metabolic pathway, quinazolines are also produced by plants and thus might reflect the dietary composition before the fecal sample was obtained. Similar relationships among the remainder of the cohort-unique metabolites could also be rationalized. 

**Figure 3:**
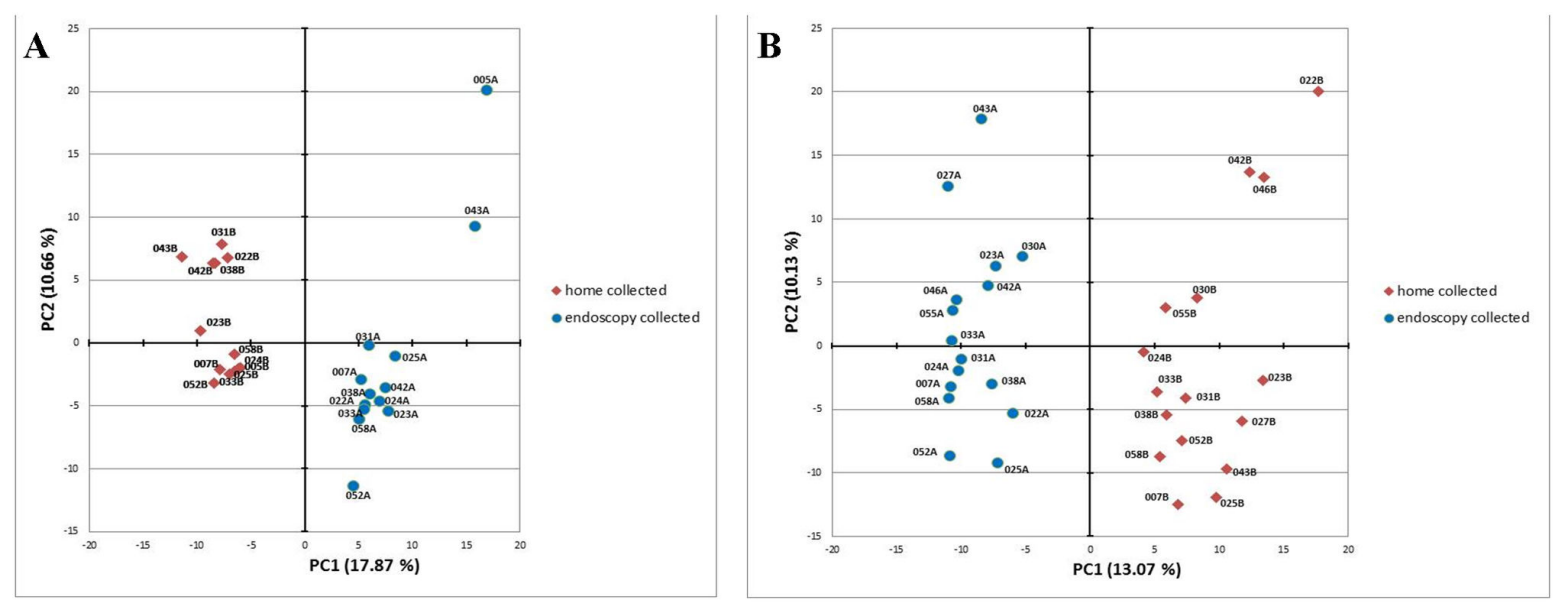
Principle component analysis of the VOC metabolomes identified in the passage and endoscopy collected human fecal samples. PCA plots derived from the identified metabolites and their abundance are presented. The first (PC1) and second (PC2) principle components are shown. In contrast to the microbiome (Figure 1), the PCA of the VOC metabolomes indicates significant differences among the home passage and endoscopy collected samples, as the samples clearly segregate according to collection technique. Analyzed were the VOC metabolomes obtained with either a 20 minute (A) or 18 hour (B) hSPME extraction. The infrequent metabolites were disregarded by restricting the analysis to analytes that appeared in a minimum of 20% of all samples in each cohort. Home collected samples are indicated as red diamonds, while endoscopy collected samples are denoted as blue circles. The naming and numbering convention of the samples is described in Figure 1. See text for further discussion.

**Figure 4:**
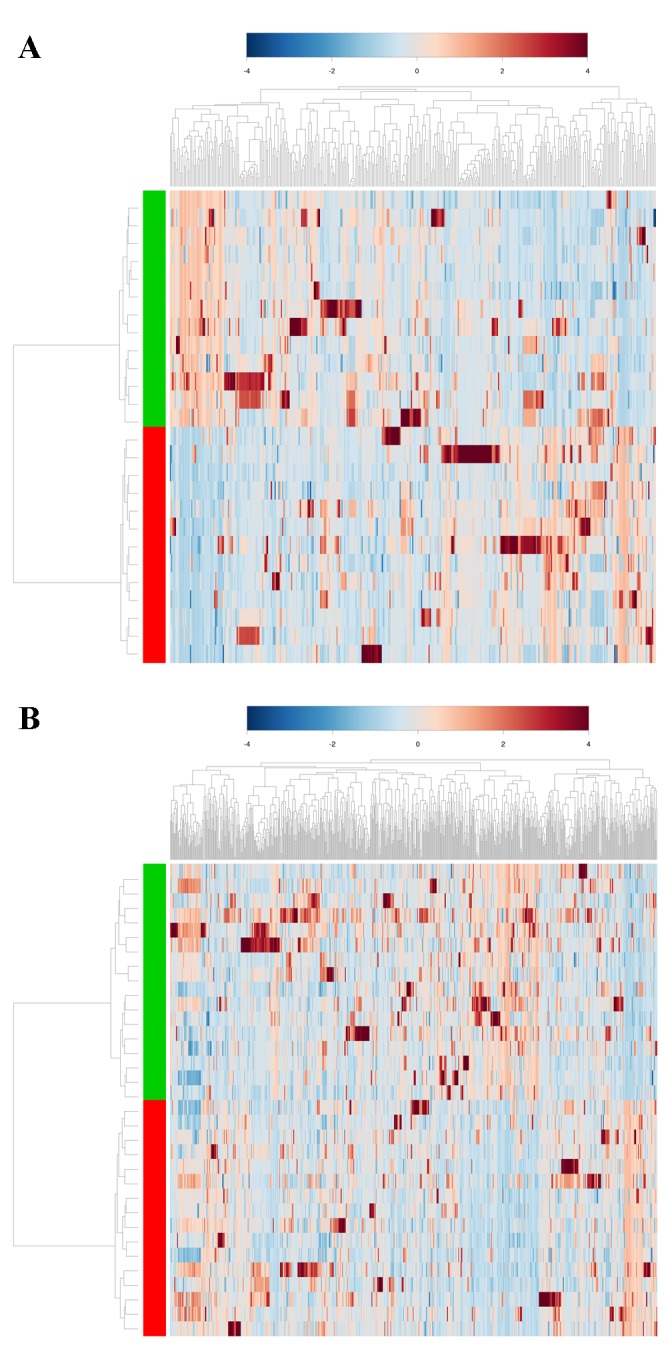
Heat map showing the unsupervised hierarchical clustering of the fecal samples according to the similarity of metabolome composition. The 20 minute metabolomes are compared in (A), while the 18 hour metabolomes are compared in (B). The samples are arranged in rows, the metabolites in columns, and shades of red represent elevation of a metabolite while shades of blue represent decrease of a metabolite relative to the median metabolite levels (see color scale). In the dendrogram, the fecal collection technique is indicated by the colored bars (green = home passage collected, red = endoscopy collected). The clustering clearly differentiates the fecal samples by collection technique.

**Figure 5:**
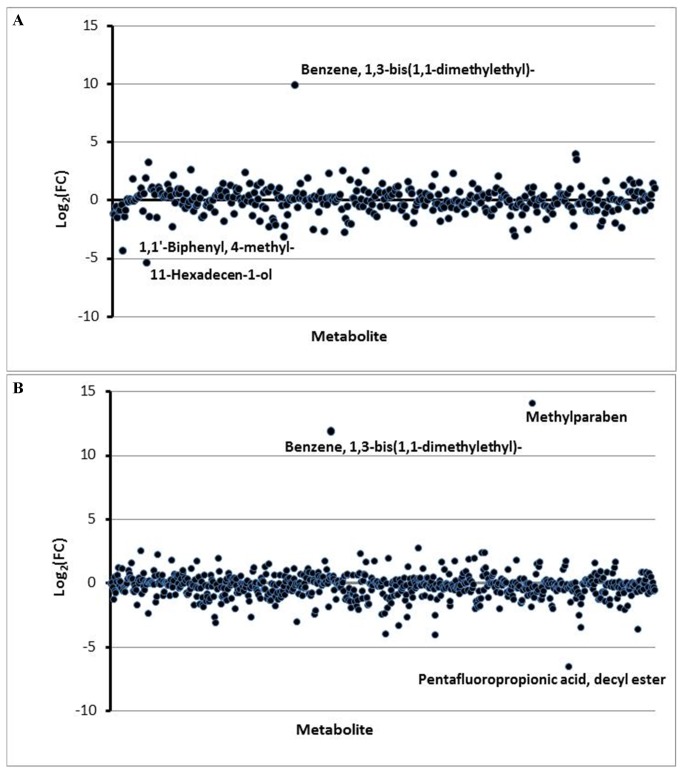
Fold change analysis of the metabolite abundance between the endoscopy and home passage collected samples. The fold change (FC) is calculated as the log transformation of the ratio between the mean metabolite abundance in the endoscopy cohort relative to the home passage collected cohort. The analysis was performed with both the 20 minute (A) and 18 hour (B) metabolomes.

While a few cohort-unique metabolites can be differentiated, the vast majority (>99%) of the identified fecal VOCs are present in both the endoscopy and home passage collected groups. To determine how the relative metabolite abundance relates among the two cohorts, a PCA was performed using the 20 minute and 18 hour metabolomes (Figure 3). In stark contrast to the relatively invariant microbiomes (Figure 1), the endoscopy collected and home passage collected samples clearly differ from each other in terms of their metabolomes, as evidenced by the samples segregating into separate cohorts on the PCA plots. With both the 20 minute and 18 hour metabolomes, the first principle component clearly discriminates between the two collection techniques, whereas the second component contributes variation within the cohorts (particularly evident with the 18 hour extraction data). Numerous metabolites collectively contribute to the segregation of the cohorts (Figure S5), the top 20 of which are identified in Figure S6. A dendrogram and accompanying heat map derived from the 20 minute and 18 hour metabolomes (Figure 4) further illustrates the clear differentiation of the endoscopy collected and home passaged collected samples, demonstrating that while the two collection cohorts are nearly identical in their specific VOC composition, changes in the abundance relationships among these metabolites differentiate the cohorts from one another. Figure 5 presents the fold change of metabolite abundance between the two cohorts. Nearly half (41%) of the total metabolome exhibits a fold change greater than 1.5. Exclusion of these ‘hypervariable’ metabolites significantly reduces the variance observed among the two cohorts, as the endoscopy collected and home passage collected cohorts no longer segregate on a PCA plot (Figure S7), clearly illustrating that the metabolome differences associated with the fecal collection technique is primarily attributed to global changes in relative metabolite abundance, rather than alterations in the specific composition of the metabolome itself. 

## Conclusions

While *in vivo* sample collection is typically desirable for metagenomic and metabolomic investigations, there are several challenges associated with the endoscopic collection of human stool, not the least of which is the significant cost compared to fecal collection at home after passage. Hence, a primary goal of our investigation was to evaluate if sample acquisition significantly biases the derived fecal microbiome and VOC metabolomes, thereby justifying the additional expense associated with endoscopic collection. While pairwise comparison of matched endoscopic and home collected samples did indeed illustrate some variability in the derived microbiomes, the vast majority (81%) of the paired samples demonstrate a strong correlation in their microbiome composition, and the two cohorts as a whole are seen to cluster together in a PCA plot, indicative of relatively little variance between them. In contrast, regardless of the hSPME extraction duration used, the VOC metabolomes differ widely between the home collected and endoscopy collected samples. While the specific VOC composition remains nearly identical, the relative abundance relationships among the metabolites were found to vary among the home and endoscopy collected samples. This is clearly depicted in Figure S8, illustrating metabolite correlation maps of the home passage collected and endoscopy collected samples. While similarities among the maps can be discerned, differences in correlation patterns are also readily distinguished. A PCA, hierarchical clustering analysis, and fold change analysis also clearly differentiate the metabolomes derived from the home and endoscopy collected samples (as detailed above), illustrating the bias of sample acquisition on the derived fecal VOC metabolome. Hence, the use of endoscopy collected samples appears justified for fecal VOC investigations (alternatively, studies evaluating methods of fecal collection and preservation are needed). 

**Figure 6:**
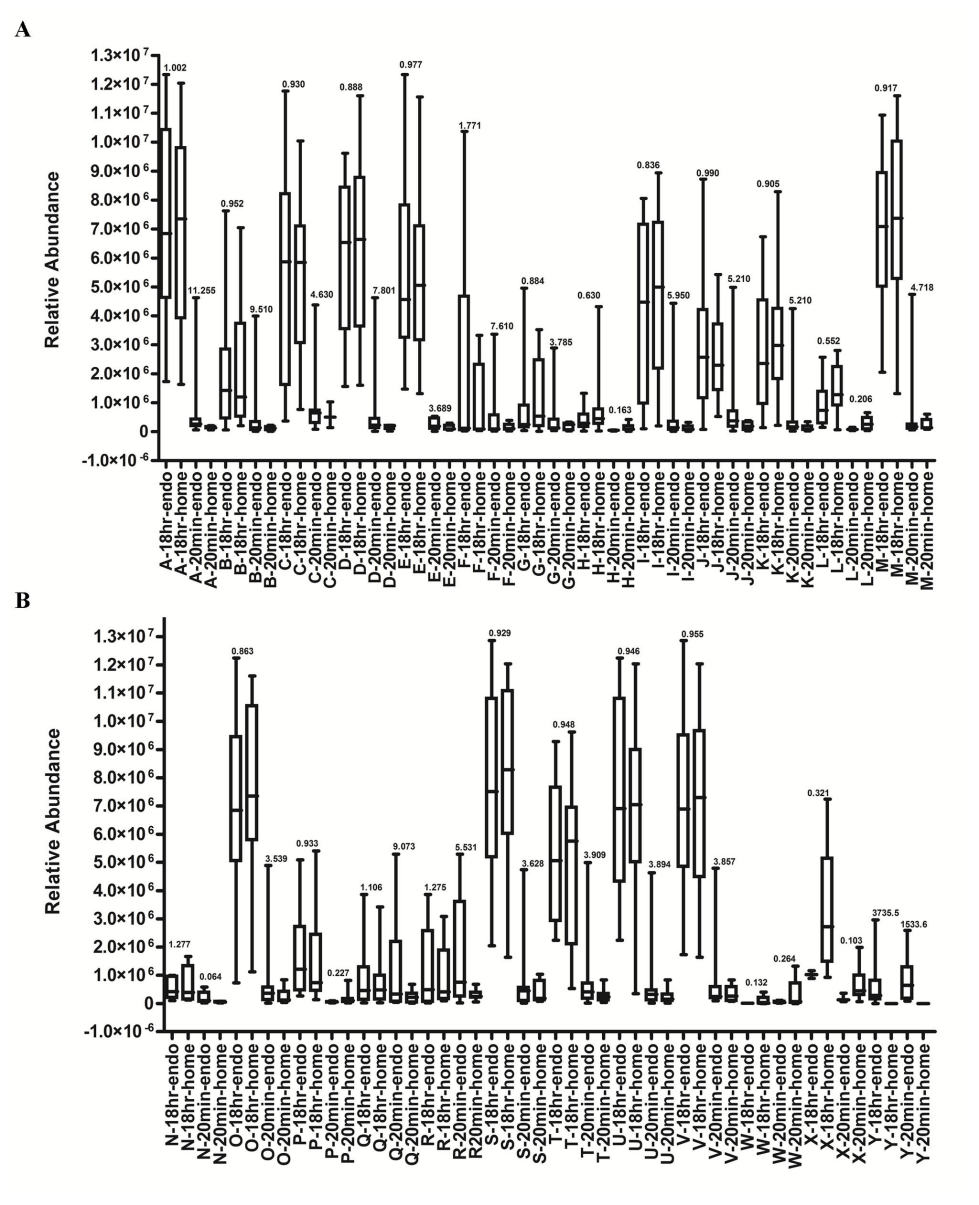
Metabolome bias introduced by short hSPME durations. The box plots illustrate the relative abundance of 25 select metabolites extracted from the home passage collected and endoscopy collected fecal samples. hSPME was performed for 20 minutes or 18 hours, as indicated. Fold change values, calculated as the log transformation of the ratio between the mean metabolite abundance in the endoscopy cohort relative to the passaged cohort, are presented over each paired endoscopy and home collection cohort. For many of the analytes, the fold change appears significant (>2) in the 20 minute metabolomes, but is deemed insignificant with the 18 hour extraction, indicative of incomplete extraction of the analyte at 20 minutes, underscoring the potential for bias associated with short, non-equilibrium extractions. Examples of analytes demonstrating insignificant fold change with a 20 minute hSPME but significant fold change with an 18 hour hSPME were also observed, but are not depicted in the Figure. The selected analytes are: A- 1-Octadecanol methyl ether; B- Dodecyl heptafluorobutyrate; C- Tridecyl acetate; D- Cyclopentane undecyl-; E- Tetracosanol-1; F- Cycloheptene, 5-ethylidene-1-methyl-; G- Benzeneacetonitrile, α-hydroxy-; H- 1-Pentanone, 1-phenyl-; I- Pentafluoropropionic acid, dodecyl ester; J- 10-Heneicosene; K- 3-Hexadecene; L- Phthalic acid, isobutyl 4-octyl ester; M- 1-Tetradecanol, methyl ether; N- Butanoic acid, methyl ester; O- Dichloroacetic acid, 4-hexadecyl ester; P- 18-Nonadecen-1-ol; Q- Menthol; R- Butanoic acid, 3,7-dimethyl-6-octenyl ester; S- 1-Tetradecyl acetate; T- 9-Nonadecene; U- 1-Heneicosyl formate; V- Octadecyl trifluoroacetate; W- Acetyl valeryl; X- Dodecalactone; Y- Benzene, 1,3-bis(1,1-dimethylethyl)-.

As the VOC extraction profile is hyperbolic, with short extraction durations more vulnerable to variation than extractions continued to equilibrium, a second goal of our investigation was to ascertain if a hSPME-based fecal metabolomics study might be biased by the extraction duration employed. In agreement with our previous observations [8], a prolonged hSPME duration (18 hours) resulted in the identification of significantly more metabolites (~750) than short (20 minute) extraction durations. Additionally, comparison of the pooled home collected metabolome with the combined endoscopy collected metabolome reveals less overall variability associated with the 18 hour extraction duration (see Figure 2). Surprisingly however, the sample to sample variation within the cohorts appears more prominent with the 18 hour extraction duration (i.e. the 20 minute extraction samples cluster more tightly in the PCA plots than do the 18 hour extraction samples), as illustrated in Figure 3 and Figure S9. While this supports the preference for a 20 minute hSPME, it is important to note that several analytes (~30) deemed unique to a cohort in the 20 minute metabolomes were found common to both the home and endoscopy collected cohorts when the VOC extraction was performed for 18 hours (Table 2). Additionally, numerous analytes perceived to have significant fold change with a 20 minute extraction duration are found insignificant with prolonged extraction (Figure 6), underscoring the potential for bias associated with a 20 minute hSPME. Hence, 18 hour hSPME appears better suited to fecal VOC metabolomics.

Our results also implicate the importance of dietary consumption on the stool VOC composition. As illustrated in Figure S4, given the geographical and cultural similarities among the majority of our study participants, we attribute nearly 50% of the entire VOC metabolome to dietary variability, with these low frequency analytes appearing in ≤20% of the total fecal samples analyzed. However, detailed and comprehensive dietary information should ideally be obtained and considered when comparing the fecal VOC profiles between cohorts involving geographic and cultural diversity, where dietary habit could be markedly different. It should also be considered that we focused our study on stool samples collected from healthy subjects and it is possible that abnormal intestinal microbiota composition (i.e. dysbiotic microbiota) in disease states might be more or less susceptible to exposure to ambient environment when the stool is collected at home. 

## Supporting Information

Figure S1 **Microbiome correlation between matched home and endoscopy collected samples.** Pearson’s correlation coefficients (r) were calculated by comparison of the microbiome content (taxa and abundance) derived from matched home and endoscopy collected samples. As depicted in the plot, 81% of the sample pairs are strongly correlated (r>0.7), whereas 19% have only weak to moderate correlation among their microbiome content (0.2<r<0.7). In contrast, comparable analysis of the derived VOC metabolomes indicates very weak (r<0.2) correlation among the samples (not shown).
(TIF)

Figure S2 **Heat map showing the unsupervised hierarchical arrangement of the fecal samples according to the similarity of microbiome composition.** The samples are arranged in rows, the taxa in columns, and shades of red represent elevation of abundance while shades of blue represent decrease abundance relative to the median values (see color scale). In the dendrogram, the fecal collection technique is indicated by the colored bars (green = home passage collected, red = endoscopy collected). The naming and numbering convention of the samples is described in Figure 1. Many of the matched sample pairs appear juxtaposed in the dendrogram, a reflection of their similarity. There is no significant segregation of the samples by fecal collection technique (in contrast to Figure 4).
(TIF)

Figure S3 **Distribution of metabolites among the endoscopy and home passage collected cohorts.** The composition of each chemical class was compared between the two cohorts and the proportion of common and unique metabolites are indicated. A) The analysis was performed using the 20 minute VOC metabolomes. A comparison of 18 hr metabolomes produced similar results (data not shown). In B) and C), infrequent metabolites were disregarded by restricting the analysis to analytes that appeared in a minimum of 20% of all samples in each cohort. The analysis was performed using the B) 20 minute extraction and C) 18 hour extraction metabolomes. The graphs in (B) have been corrected to account for the observation that several analytes identified as unique in the 20 minute extraction data set were actually found to be common to both cohorts after prolonged (18 hour) extraction. The inverse was not found to occur.
(TIF)

Figure S4 **Number of identified VOCs as a function of frequency of appearance among the total number of fecal samples analyzed.** A) Distribution in the 20 minute metabolome. B) Distribution in the 18 hr metabolome. In both cases, a large number of analytes appear in a small number of fecal samples, likely a reflection of dietary variation among the study participants.
(TIF)

Figure S5 **Metabolite contribution to the first and second principle components.** The contribution to the first principle component (squared cosine of the variable) by the top 100 contributing analytes is presented for the A) 20 minute and B) 18 hour metabolomes. Corresponding contributions to the second principle component are shown in C) and D), respectively.
(TIF)

Figure S6 **Biplots of the top 20 metabolites with the greatest loadings in the PCAs.** Plots for the A) 20 minute and B) 18 hour metabolomes are shown.
(TIF)

Figure S7 **Principle component analysis of the VOC metabolomes identified in the home passage and endoscopy collected human fecal samples, with the omission of the metabolites with a fold change greater than 1.5.** The first (PC1) and second (PC2) principle components are shown. In contrast to Figure 3, the samples no longer segregate according to collection technique, underscoring the importance of the relationship between fecal collection technique and the relative abundance of the metabolites.
(TIF)

Figure S8 **Metabolite correlation maps of endoscopy collected (left) and home passage collected (right) 18 hour VOC metabolomes.** Metabolites are arranged along the horizontal and vertical axes and the Pearson correlation values are depicted in the heat map. Shades of red represent positive correlation among the metabolites while shades of green represent negative correlation (see color scale). The two correlation maps have notable differences.
(TIF)

Figure S9 **Principle component analysis of the VOC metabolomes derived from the home passage collected human fecal samples.** PCA plots reflect the identified metabolites and their abundance obtained with either a 20 minute or 18 hour hSPME. The analysis was restricted to analytes appearing in a minimum of A) 20% of all samples in each cohort or B) 80% of all samples in each cohort. In either case, the 20 minute metabolomes clearly segregate from the 18 hour metabolomes and the 20 minute extraction samples cluster more tightly than do the 18 hour extraction samples. The naming and numbering convention of the samples is described in Figure 1.
(TIF)

## References

[1] Tremaroli V, Bäckhed F (2012) Functional interactions between the gut microbiota and host metabolism. Nature 489: 242–249. doi:10.1038/nature11552. PubMed: 22972297. 22972297

[2] Russell SL, Finlay BB (2012) The impact of gut microbes in allergic diseases. Curr Opin Gastroenterol 28: 563–569. doi:10.1097/MOG.0b013e3283573017. PubMed: 23010680. 23010680

[3] Ivanov II, Honda K (2012) Intestinal commensal microbes as immune modulators. Cell Host Microbe 12: 496–508. doi:10.1016/j.chom.2012.09.009. PubMed: 23084918. 23084918 PMC3516493

[4] Garner CE, Smith S, De Lacy Costello B, White P, Spencer R et al. (2007) Volatile organic compounds from feces and their potential for diagnosis of gastrointestinal disease. FASEB J 21: 1675–1688. doi:10.1096/fj.06-6927com. PubMed: 17314143. 17314143

[5] De Lacy Costello B (2008) An analysis of volatiles in the headspace of the faeces of neonates. J Breath Res 2: 037023. doi:10.1088/1752-7155/2/3/037023. PubMed: 21386183. 21386183

[6] Garner CE, Smith S, Bardhan PK, Ratcliffe NM, Probert CS (2009) A pilot study of faecal volatile organic compounds in faeces from cholera patients in Bangladesh to determine their utility in disease diagnosis. Trans R Soc Trop Med Hyg 103: 1171–1173. doi:10.1016/j.trstmh.2009.02.004. PubMed: 19268999. 19268999

[7] Garner CE, Ewer AK, Elasouad K, Power F, Greenwood R et al. (2009) Analysis of faecal volatile organic compounds in preterm infants who develop necrotising enterocolitis: a pilot study. J Pediatr Gastroenterol Nutr 49: 559–565. doi:10.1097/MPG.0b013e3181a3bfbc. PubMed: 19668005. 19668005

[8] Dixon E, Clubb C, Pittman S, Ammann L, Rasheed Z et al. (2011) Solid-phase microextraction and the human fecal VOC metabolome. PLOS ONE 6: e18471. doi:10.1371/journal.pone.0018471. PubMed: 21494609. 21494609 PMC3072975

[9] Boland W (1984). nalysis of Volatiles: 371-380.

[10] Schulz S, Fuhlendorff J, Reichenbach H (2004) Identification and synthesis of volatiles released by the myxobacterium Chondromyces crocatus. Tetrahedron 60: 3863–3872. doi:10.1016/j.tet.2004.03.005.

[11] Arthur CL, Pawliszyn J (1990) Solid-Phase Microextraction with Thermal-Desorption Using Fused-Silica Optical Fibers. Anal Chem 62: 2145–2148. doi:10.1021/ac00218a019.

[12] Alpendurada MD (2000) Solid-phase microextraction: a promising technique for sample preparation in environmental analysis. J Chromatogr A 889: 3–14. doi:10.1016/S0021-9673(00)00453-2. PubMed: 10985530. 10985530

[13] Li RW, Wu S, Li W, Navarro K, Couch RD et al. (2012) Alterations in the Porcine Colon Microbiota Induced by the Gastrointestinal Nematode Trichuris suis. Infect Immun 80: 2150–2157. doi:10.1128/IAI.00141-12. PubMed: 22493085. 22493085 PMC3370577

[14] Pawliszyn J (1999) Quantitative aspects of SPME. Cambridge: the Royal Society of Chemistry.

[15] Koziel J, Jia M, Pawliszyn J (2000) Air sampling with porous solid-phase microextraction fibers. Anal Chem 72: 5178–5186. doi:10.1021/ac000518l. PubMed: 11080861. 11080861

[16] Roediger WE (1980) Anaerobic bacteria, the colon and colitis. Aust N Z J Surg 50: 73–75. doi:10.1111/j.1445-2197.1980.tb04500.x. PubMed: 6928766. 6928766

[17] Vedantam G, Hecht DW (2003) Antibiotics and anaerobes of gut origin. Curr Opin Microbiol 6: 457–461. doi:10.1016/j.mib.2003.09.006. PubMed: 14572537. 14572537

[18] Gillevet P, Sikaroodi M, Keshavarzian A, Mutlu EA (2010) Quantitative Assessment of the Human Gut Microbiome Using Multitag Pyrosequencing. Chem Biodivers 7: 1065–1075. doi:10.1002/cbdv.200900322. PubMed: 20491064. 20491064 PMC3748609

[19] Xia J, Mandal R, Sinelnikov IV, Broadhurst D, Wishart DS (2012) MetaboAnalyst 2.0—a comprehensive server for metabolomic data analysis. Nucleic Acids Res vol 40: w127-w133. doi:10.1093/nar/gks374. PubMed: 22553367. 22553367 PMC3394314

[20] Kawamura Y (2004) Effects of Gamma Irradiation on Polyethylene, Polypropylene, and Polystyrene. In: Komolprasert V Morehouse KM Irradiation of Food and Packaging, Vol. 875 Washington, DC: American Chemical Society pp. 262–276.

[21] Ratel J, Engel E (2009) Determination of benzenic and halogenated volatile organic compounds in animal-derived food products by one-dimensional and comprehensive two-dimensional gas chromatography–mass spectrometry. J Chromatogr A 1216: 7889–7898. doi:10.1016/j.chroma.2009.09.012. PubMed: 19782373. 19782373

[22] Lou F, Li Q, Qiu W (2011) Analysis on the essential oil of Magnolia officinalis Rehd. et Wils. in five different habitats by GC-MS. Anhui Nongye Kexue 39: 3934–3937..

[23] Xing S, Zhang P, Ji Q, Jia H, Wang X (2010) Essential oil compositions and antioxidant activities of two Ziziphora species in Xinjiang. Shipin Kexue (Beijing, China) 31: 154–159

[24] Huo X, Gao Y, Yang N, Liu W, Liu J (2008) Chemical composition of the essential oil of Herba Taxilli. Shengwu Jishu 18: 47–49.

[25] Economou D, Nahrstedt A (1991) Chemical, physiological, and toxicological aspects of the essential oil of some species of the genus Bystropogon. Planta Med 57: 347–351. doi:10.1055/s-2006-960115. PubMed: 1775576. 1775576

[26] Moyler D, Moss N (1998) Mint oils: potential for standardizing profiles with natural flavoring substances. Perfumer Flavorist 23: 37–42.

[27] Hashim L, Hudiyono S, Chaveron H (1997) Volatile compounds of oxidized cocoa butter. Food Res Int 30: 163–169. doi:10.1016/S0963-9969(97)00039-2.

[28] Gómez E, Ledbetter CA (1997) Development of Volatile Compounds during Fruit Maturation: Characterization of Apricot and Plum×Apricot Hybrids. J Sci Food Agric 74: 541–546. doi:10.1002/(SICI)1097-0010(199708)74:4.

[29] Georgilopoulos DN, Gallois AN (1987) Aroma compounds of fresh blackberries (Rubus laciniata L.). Zeitschrift fr Lebensmittel-Untersuchung und -Forschung 184: 374–380. 10.1007/BF01123035 3425000

[30] Dickschat JS, Martens T, Brinkhoff T, Simon M, Schulz S (2005) Volatiles released by a Streptomyces species isolated from the North Sea. Chem Biodivers 2: 837–865. doi:10.1002/cbdv.200590062. PubMed: 17193176. 17193176

[31] Cox CD, Parker J (1979) Use of 2-aminoacetophenone production in identification of Pseudomonas aeruginosa. J Clin Microbiol 9: 479–484. PubMed: 110829. 110829 10.1128/jcm.9.4.479-484.1979 PMC273058

[32] Mann S (1967) [Quinazoline derivatives in pseudomonads]. Arch Mikrobiol 56: 324–329. doi:10.1007/BF00425207. PubMed: 4970237. 4970237

