# Supplementary figures and images for: The Approach to Sample Acquisition and Its Impact on the Derived Human Fecal Microbiome and VOC Metabolome
Source: PLoS One. 2013 Nov 18;8(11):e81163. doi: 10.1371/journal.pone.0081163 (PMC3832442; doi:10.1371/journal.pone.0081163)

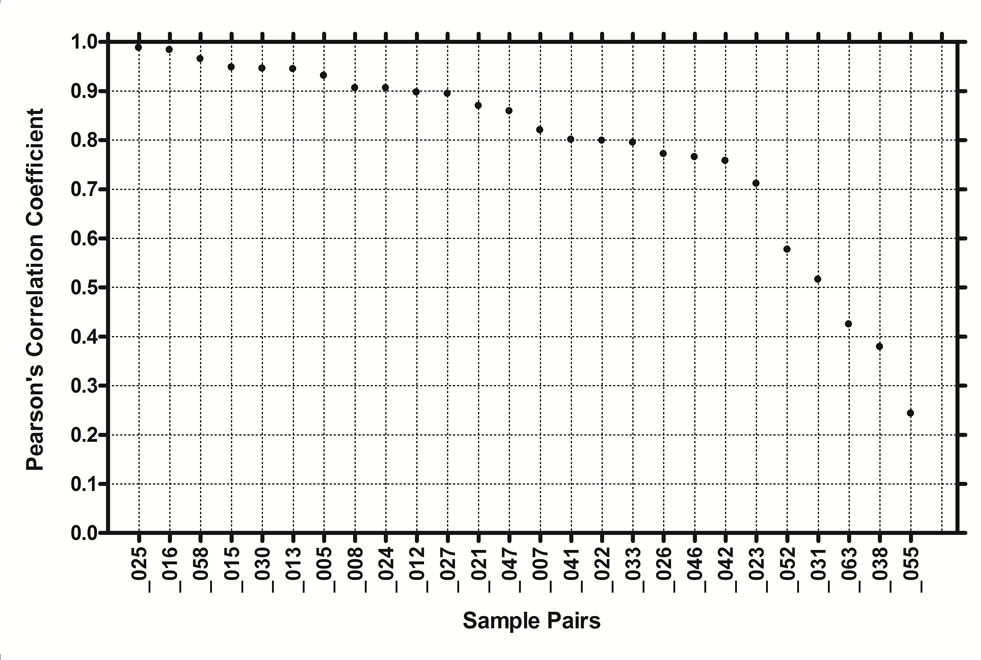

Supplement: Figure S1 — Microbiome correlation between matched home and endoscopy collected samples. Pearson’s correlation coefficients (r) were calculated by comparison of the microbiome content (taxa and abundance) derived from matched home and endoscopy collected samples. As depicted in the plot, 81% of the sample pairs are strongly correlated (r>0.7), whereas 19% have only weak to moderate correlation among their microbiome content (0.2<r<0.7). In contrast, comparable analysis of the derived VOC metabolomes indicates very weak (r<0.2) correlation among the samples (not shown). (TIF) [file pone.0081163.s001.tif]

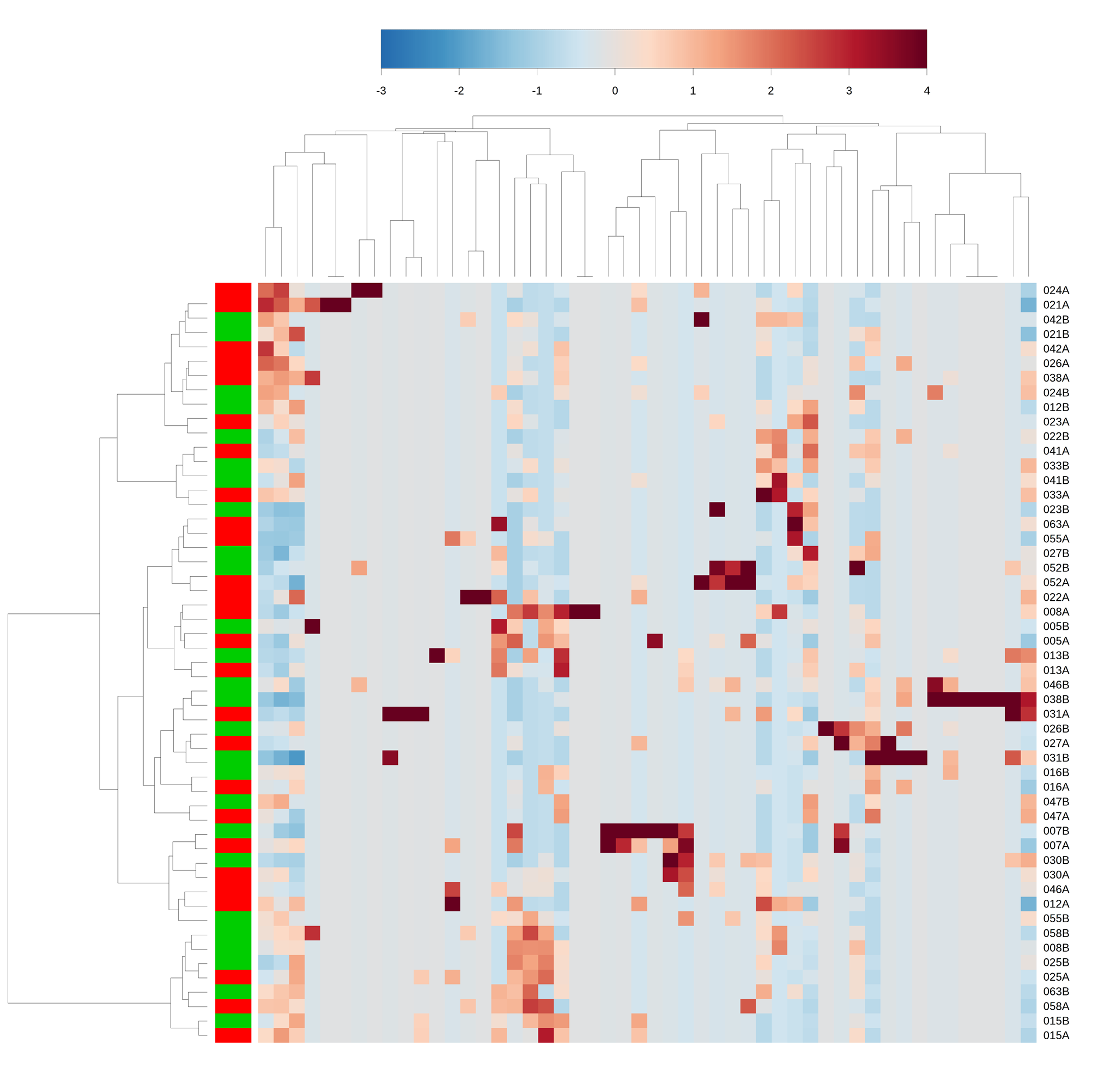

Supplement: Figure S2 — Heat map showing the unsupervised hierarchical arrangement of the fecal samples according to the similarity of microbiome composition. The samples are arranged in rows, the taxa in columns, and shades of red represent elevation of abundance while shades of blue represent decrease abundance relative to the median values (see color scale). In the dendrogram, the fecal collection technique is indicated by the colored bars (green = home passage collected, red = endoscopy collected). The naming and numbering convention of the samples is described in Figure 1. Many of the matched sample pairs appear juxtaposed in the dendrogram, a reflection of their similarity. There is no significant segregation of the samples by fecal collection technique (in contrast to Figure 4). (TIF) [file pone.0081163.s002.tif]

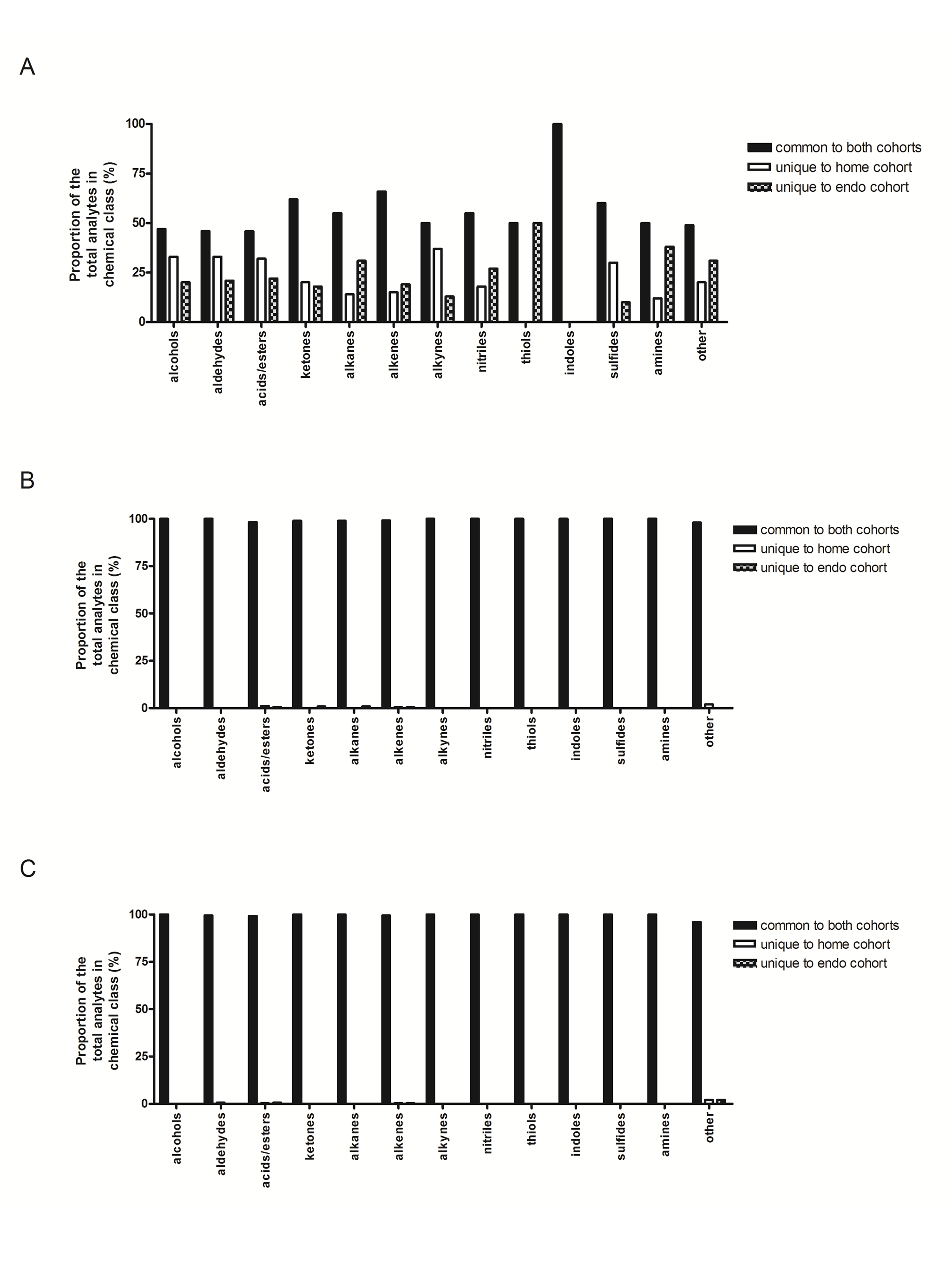

Supplement: Figure S3 — Distribution of metabolites among the endoscopy and home passage collected cohorts. The composition of each chemical class was compared between the two cohorts and the proportion of common and unique metabolites are indicated. A) The analysis was performed using the 20 minute VOC metabolomes. A comparison of 18 hr metabolomes produced similar results (data not shown). In B) and C), infrequent metabolites were disregarded by restricting the analysis to analytes that appeared in a minimum of 20% of all samples in each cohort. The analysis was performed using the B) 20 minute extraction and C) 18 hour extraction metabolomes. The graphs in (B) have been corrected to account for the observation that several analytes identified as unique in the 20 minute extraction data set were actually found to be common to both cohorts after prolonged (18 hour) extraction. The inverse was not found to occur. (TIF) [file pone.0081163.s003.tif]

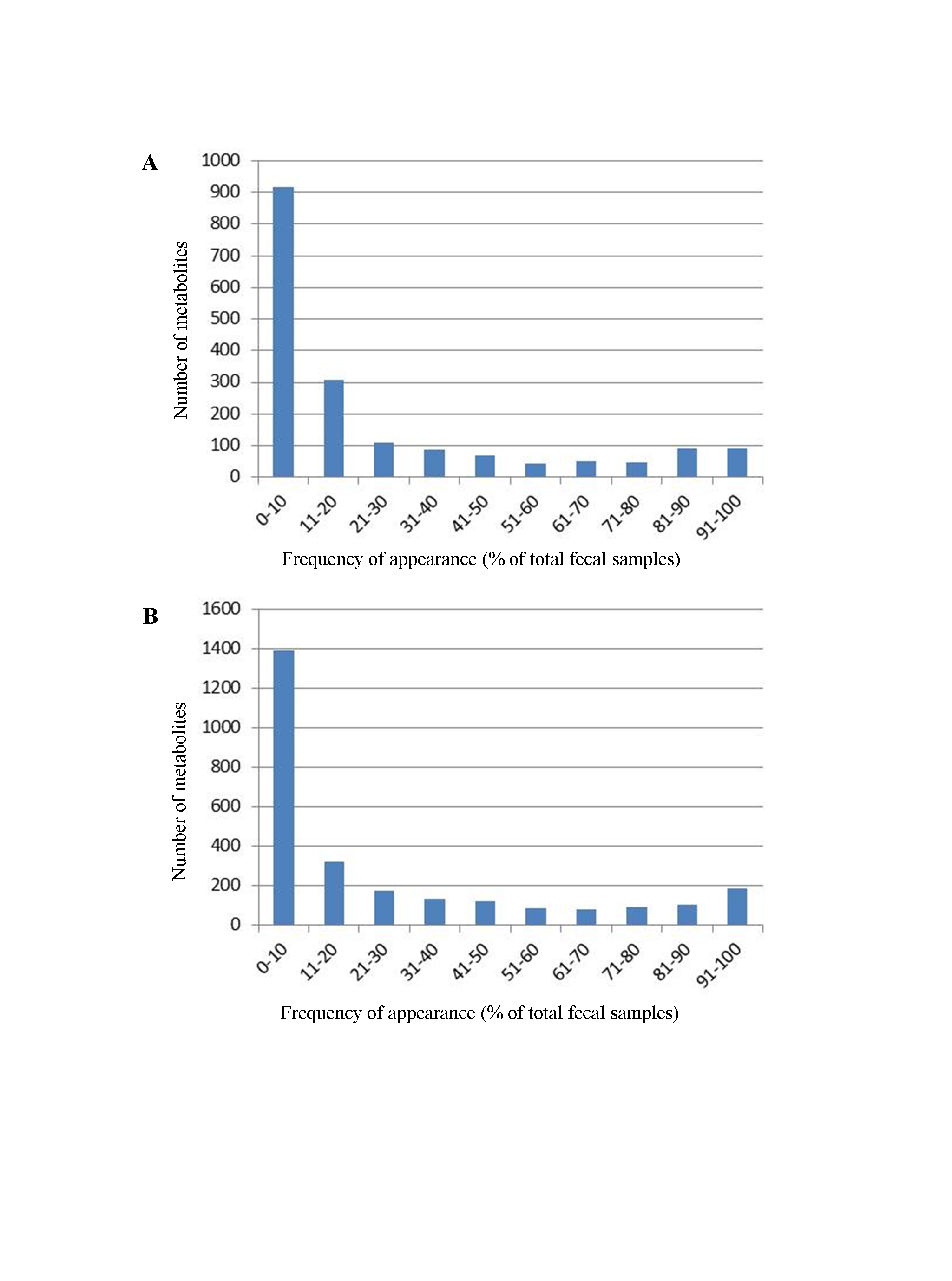

Supplement: Figure S4 — Number of identified VOCs as a function of frequency of appearance among the total number of fecal samples analyzed. A) Distribution in the 20 minute metabolome. B) Distribution in the 18 hr metabolome. In both cases, a large number of analytes appear in a small number of fecal samples, likely a reflection of dietary variation among the study participants. (TIF) [file pone.0081163.s004.tif]

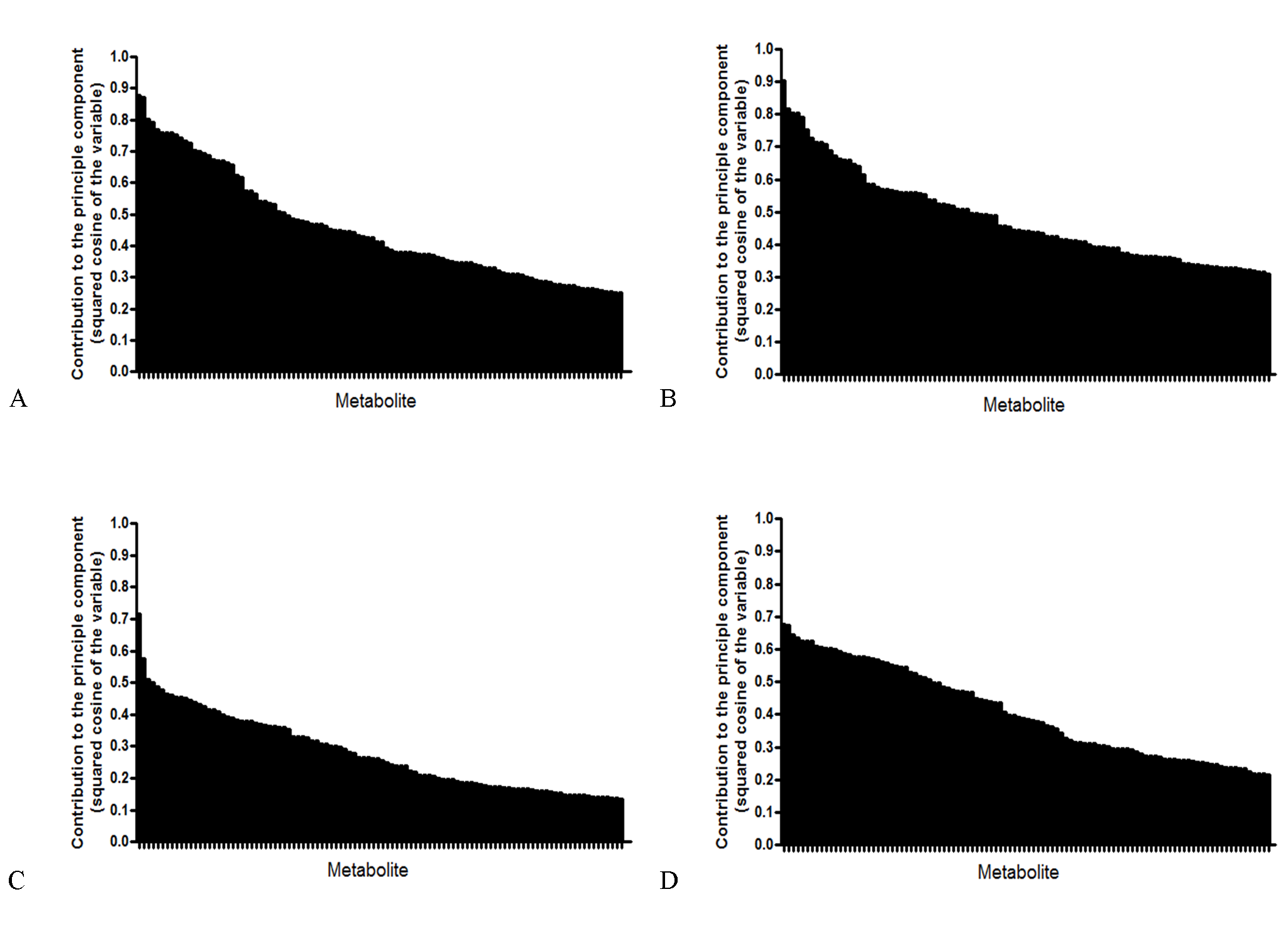

Supplement: Figure S5 — Metabolite contribution to the first and second principle components. The contribution to the first principle component (squared cosine of the variable) by the top 100 contributing analytes is presented for the A) 20 minute and B) 18 hour metabolomes. Corresponding contributions to the second principle component are shown in C) and D), respectively. (TIF) [file pone.0081163.s005.tif]

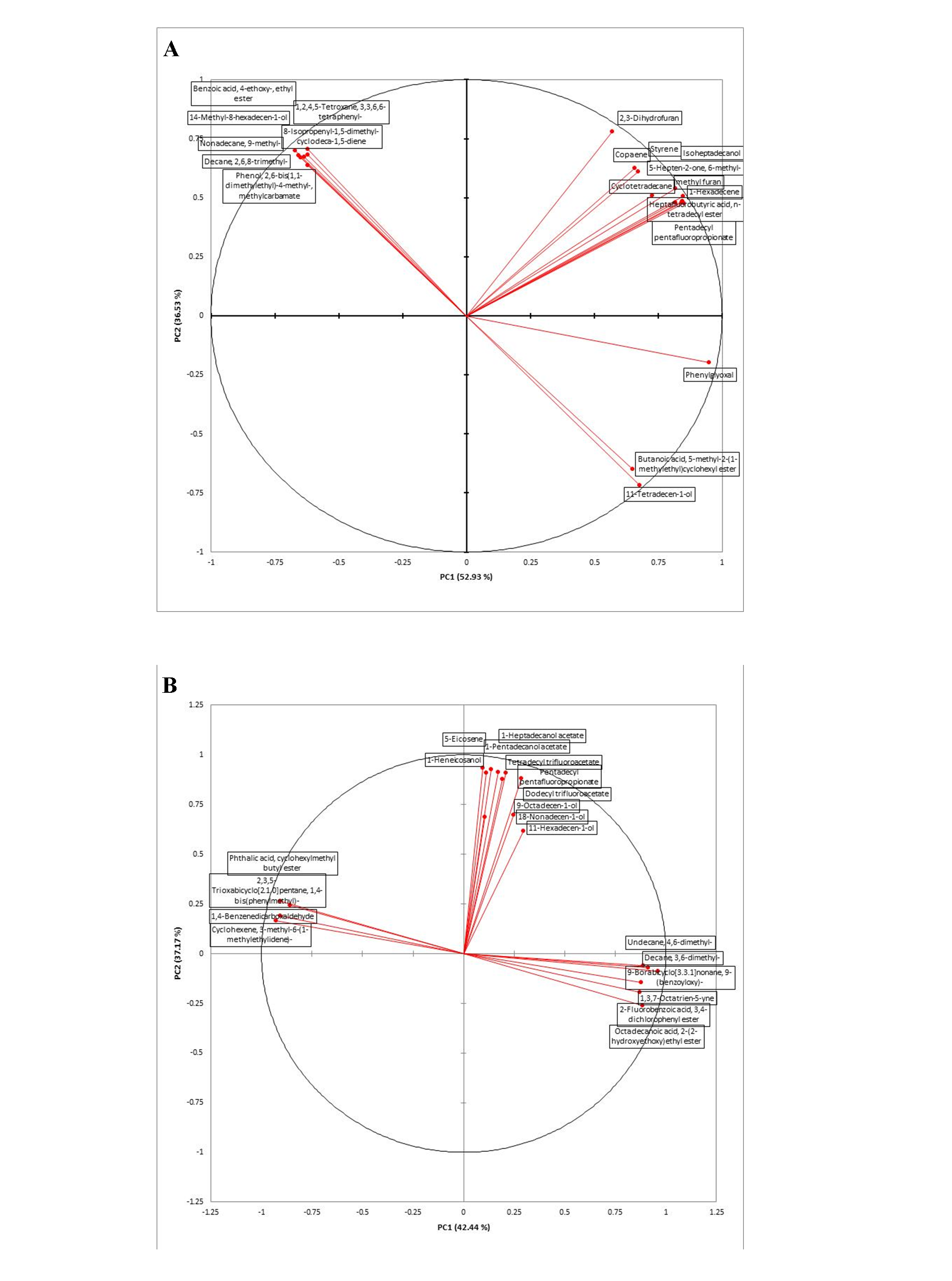

Supplement: Figure S6 — Biplots of the top 20 metabolites with the greatest loadings in the PCAs. Plots for the A) 20 minute and B) 18 hour metabolomes are shown. (TIF) [file pone.0081163.s006.tif]

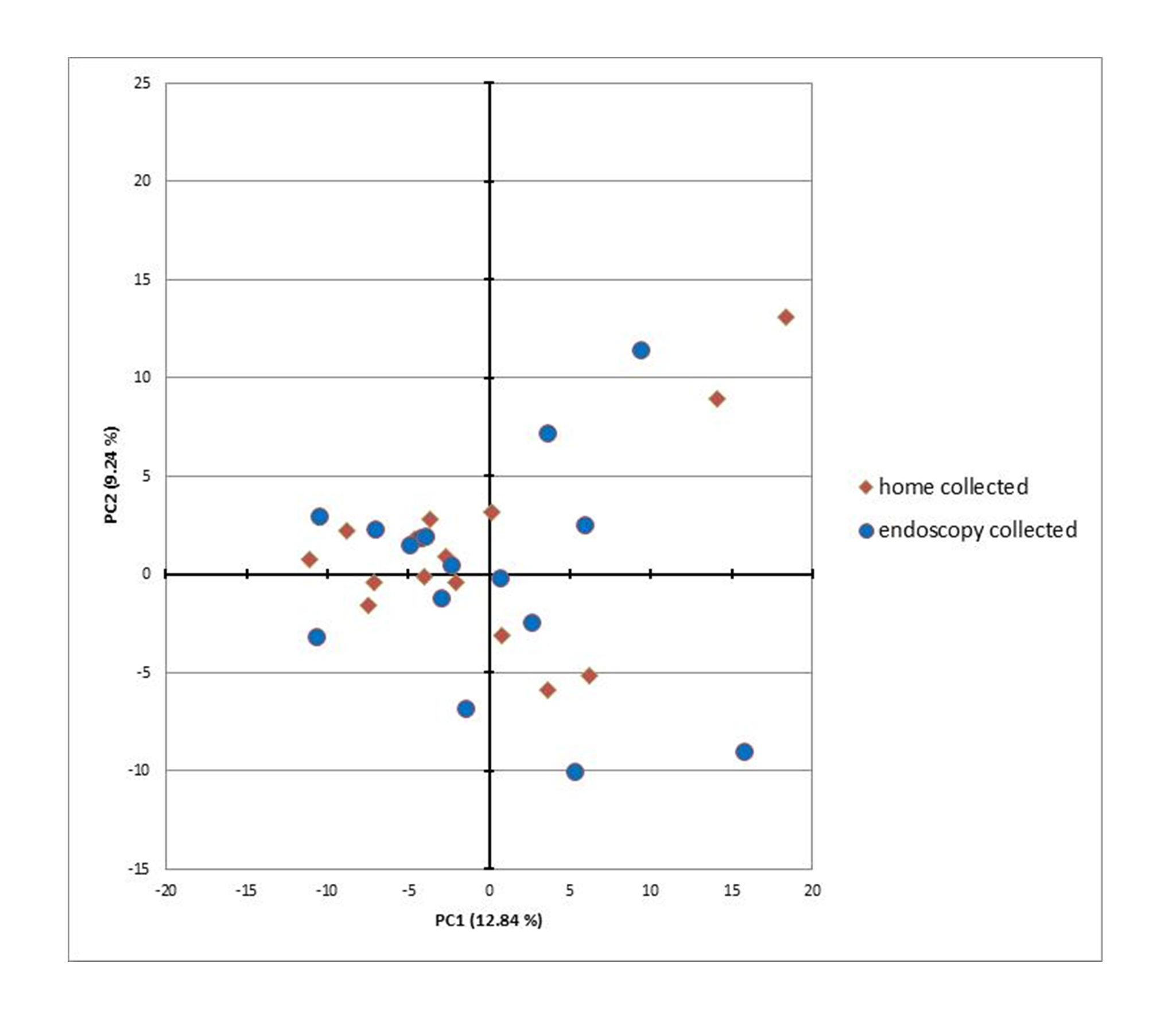

Supplement: Figure S7 — Principle component analysis of the VOC metabolomes identified in the home passage and endoscopy collected human fecal samples, with the omission of the metabolites with a fold change greater than 1.5. The first (PC1) and second (PC2) principle components are shown. In contrast to Figure 3, the samples no longer segregate according to collection technique, underscoring the importance of the relationship between fecal collection technique and the relative abundance of the metabolites. (TIF) [file pone.0081163.s007.tif]

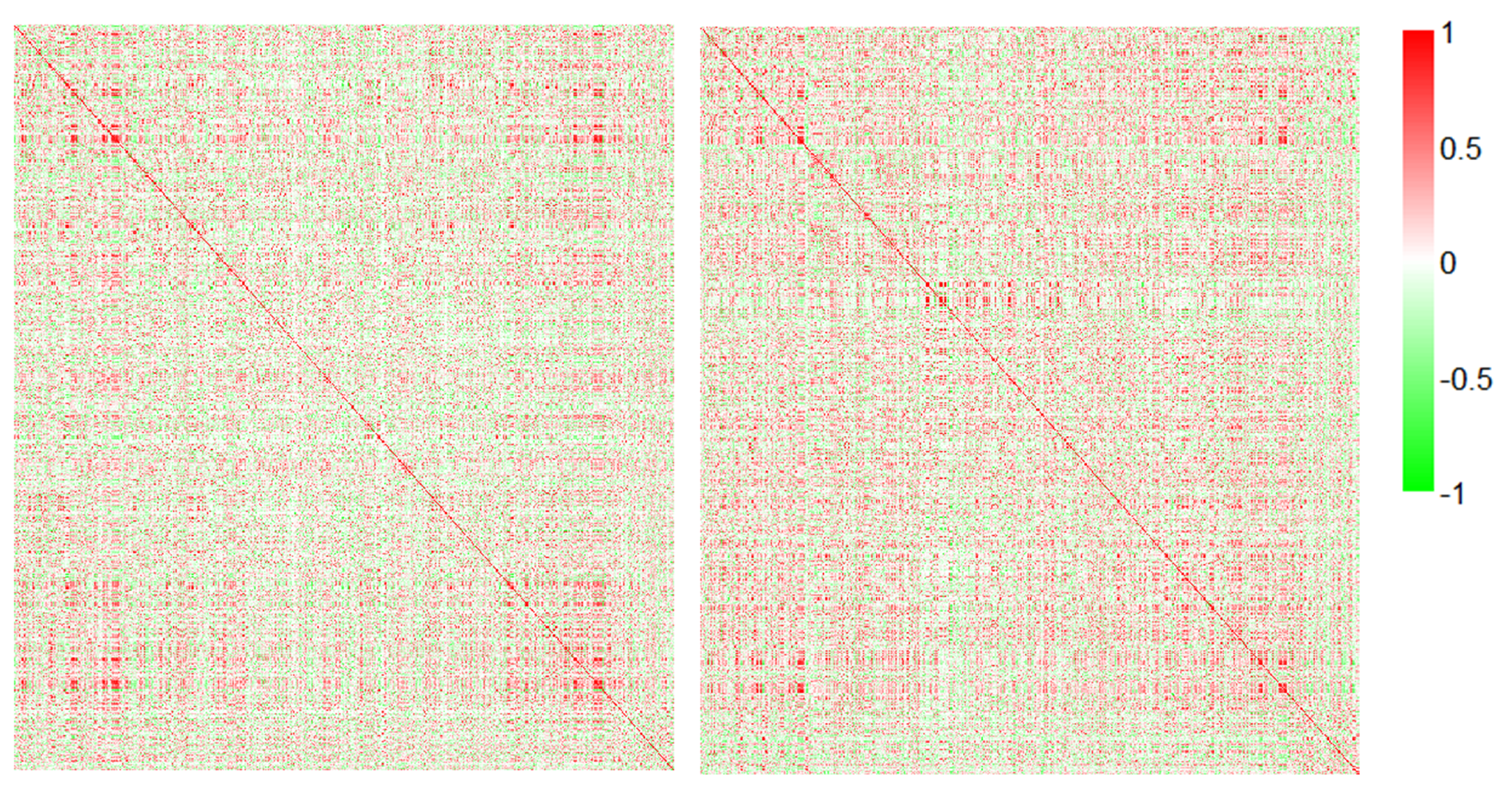

Supplement: Figure S8 — Metabolite correlation maps of endoscopy collected (left) and home passage collected (right) 18 hour VOC metabolomes. Metabolites are arranged along the horizontal and vertical axes and the Pearson correlation values are depicted in the heat map. Shades of red represent positive correlation among the metabolites while shades of green represent negative correlation (see color scale). The two correlation maps have notable differences. (TIF) [file pone.0081163.s008.tif]

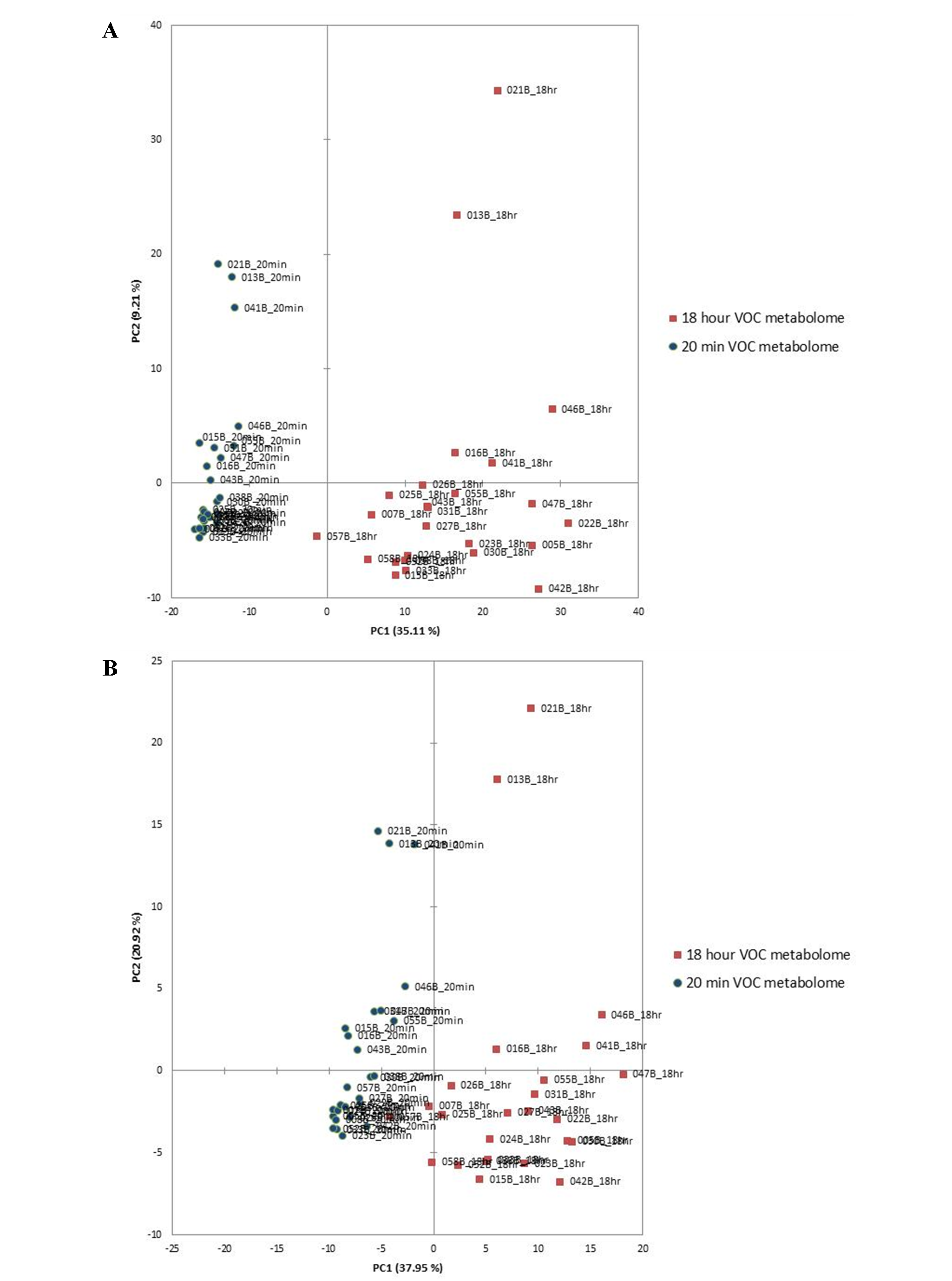

Supplement: Figure S9 — Principle component analysis of the VOC metabolomes derived from the home passage collected human fecal samples. PCA plots reflect the identified metabolites and their abundance obtained with either a 20 minute or 18 hour hSPME. The analysis was restricted to analytes appearing in a minimum of A) 20% of all samples in each cohort or B) 80% of all samples in each cohort. In either case, the 20 minute metabolomes clearly segregate from the 18 hour metabolomes and the 20 minute extraction samples cluster more tightly than do the 18 hour extraction samples. The naming and numbering convention of the samples is described in Figure 1. (TIF) [file pone.0081163.s009.tif]
